# Anatomic Thoracoscopic Repair of Esophageal Atresia

**DOI:** 10.3389/fped.2016.00142

**Published:** 2017-01-09

**Authors:** Joana Fonte, Catarina Barroso, Ruben Lamas-Pinheiro, Ana R. Silva, Jorge Correia-Pinto

**Affiliations:** ^1^Life and Health Sciences Research Institute (ICVS), School of Medicine, University of Minho, Braga, Portugal; ^2^ICVS/3B’s – PT Government Associate Laboratory, Braga/Guimarães, Portugal; ^3^Department of Pediatric Surgery, Hospital de Braga, Braga, Portugal

**Keywords:** esophageal atresia, tracheoesophageal fistula, azygos vein, minimally invasive surgery, neonatal thoracoscopy

## Abstract

**Background:**

The thoracoscopic approach to repair esophageal atresia (EA) with tracheoesophageal fistula (TEF) provides excellent view, allowing the most skillful surgeons to spare the azygos vein by performing the esophageal anastomosis over (on the right side) the azygos vein. Seeking the most anatomic repair, we started to perform the esophageal anastomosis underneath (on the left side) the azygos vein: anatomic thoracoscopic repair of esophageal atresia (ATREA). We aim to compare results of ATREA with the classic thoracoscopic repair.

**Methods:**

During the last 4 years, in our center, all infants with EA with distal TEF were operated by thoracoscopy sparing the azygos vein. According to the surgical technique, two groups were created: Group A—treated with ATREA and Group B—treated with classic thoracoscopic repair over (on the right side) the azygos vein. We retrospectively collected data regarding features of the newborn (gestational age, gender, karyotype changes, associated anomalies, birth weight), surgery (operative technique, operative time, and surgical complications), hospitalization (duration of mechanical ventilation, thoracic drainage, time for the first feeding, time of admission, and early complications) and follow-up [tracheomalacia, gastroesophageal reflux disease (GERD), anastomotic stricture, recurrence of fistula].

**Results:**

Group A had seven newborns and Group B had four newborns. There were no statistically significant differences between both groups concerning the evaluated variables on surgery, hospitalization, and follow-up. Nevertheless, in Group A, there was an infant with a right aortic arch where ATREA was particularly useful as it avoided that the azygos vein and the aortic arch were left compressed in between the esophagus and trachea. Postoperatively, one patient of Group B had a major anastomotic leak with empyema requiring surgical re-intervention. During follow-up, anastomotic stricture requiring esophageal dilation occurred with similar rates in both groups. In Group B, one patient had severe and symptomatic tracheomalacia requiring aortopexy and severe GERD requiring fundoplication. No patients developed recurrent fistula.

**Conclusion:**

The ATREA is feasible in the great majority of patients with EA with TEF without compromising long-term results and might be particularly useful for those infants with malformations of the cardiac venous return vessels and/or major aortic malformations.

## Introduction

Inspired by the well-documented benefits of minimally invasive surgery (MIS) in adults and children, pioneers of pediatric MIS have recognized the potential of thoracoscopic approach to repair the esophageal atresia with tracheoesophageal fistula. During last decade, the thoracotomy approach, refined during almost 100 years (1, 2), is being replaced by thoracoscopic approach for surgical correction of EA (3–8), even some concerns had been raised about thoracoscopy in neonates (9).

This has become the standard approach in many centers, since it allows a better cosmetic result, avoids the musculoskeletal sequelae of thoracotomy, and allows excellent magnified visualization of anatomic structures on the monitor.

Either by thoracotomy or thoracoscopy, the standard technique to correct EA with TEF is performed with the ligation and division of azygos vein.

Recently, preservation of azygos vein has been hypothesized to maintain the mediastinal venous drainage, thereby decreasing postoperative chest congestion and tissue edema, thus working as an additional protective factor against postoperative complications like anastomotic leak (10, 11), but this hypothesis was never hemodynamically confirmed. More recently, Patkowski et al. performed thoracoscopic repair sparing the azygos vein as a strategy to reduce the fistula recurrence by interposing some natural biological tissue between the anastomosis and fistula stump (12). We questioned if the azygos vein would be able to keep its patency in this position. On the other side, we know that patency of the azygos vein is important to preserve to normal hemodynamics in particularly cases (13).

In this line of thought, we hypothesized if fully anatomic thoracoscopic repair of esophageal atresia (ATREA) would be feasible without compromising outcomes. This study presents ATREA technique for the repair of EA with TEF and retrospectively reviews its results comparing with the classic thoracoscopic approach.

## Materials and Methods

Newborns with the diagnosis of EA with TEF, submitted to thoracoscopic repair between September 2012 and September 2016, were operated by the same surgeon (JCP) and included in this study. Patients submitted to open approach or with pure EA were excluded.

This study was approved by the “Comissão de Ética para o Hospital de Braga (ref. CESHB 087/2016)” and “Subcomissão de Ética para as Ciências da Vida e da Saúde (ref. SECVS 015/2016),” and the patients were de-identified to protect their anonymity.

Since 2013, we started to perform the esophageal anastomosis underneath (on the left side) the azygos vein: ATREA. The newborns were divided in two groups according to the surgical technique being performed: ATREA in Group A (patients operated more recently) and the classic thoracoscopic repair sparing the azygos vein in Group B (patients operated until 2013). In the case of technical difficulties, the ATREA was converted in the classical thoracoscopic repair sparing the azygos vein and integrated into the Group B as it occurred in one case when we realized that the anastomosis would be left with moderate to severe tension.

Data were collected regarding the prenatal period (gestational age and prenatal diagnosis), features of the newborn (gender, karyotype changes, associated anomalies, birth weight), surgery (age at the time of surgery, operative time, surgery complications), hospitalization (duration of mechanical ventilation, hospitalization and thoracic drainage, time to first feeding, and early postoperative complications), and follow-up (long-term complications such as fistula recurrence, leak and/or anastomotic stenosis, tracheomalacia, gastroesophageal reflux disease (GERD), and mortality).

Statistical analysis was performed using IMB SPSS Statistics 23. Mann–Whitney *U* test and Fisher’s exact test were used for the small sample size. Statistical significance was set at *p*-value of ≤0.05.

### Perioperative Approach and Surgical Technique

Clinical features of EA, unsuccessful orogastric intubation and thoracic roentgenogram confirmed the diagnosis. Prior to the surgical intervention, an echocardiogram was performed to identify cardiac and vascular abnormalities.

**Figure 1:**
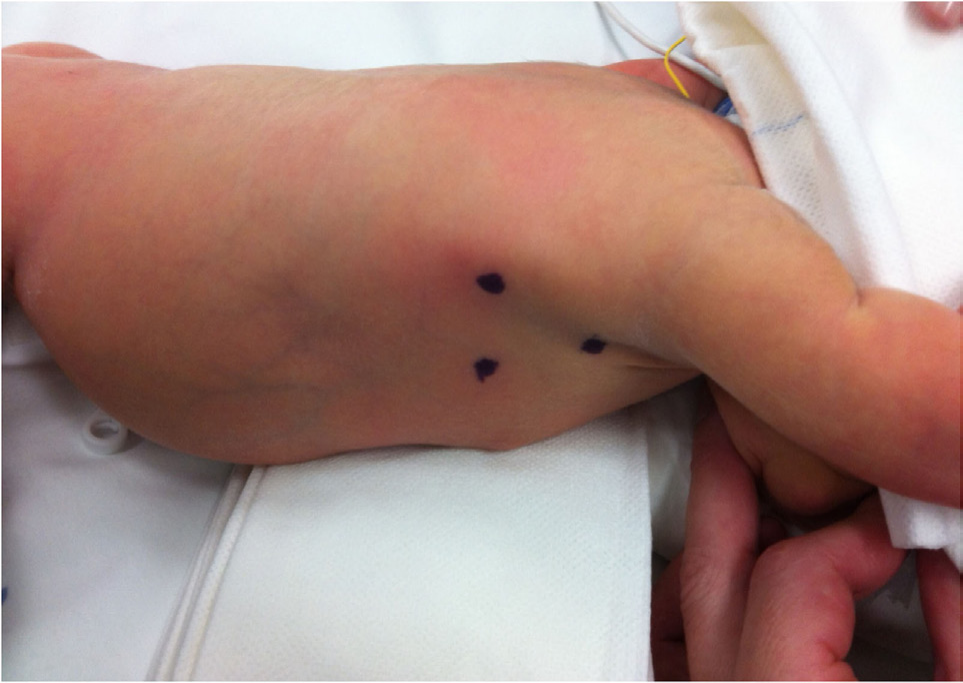
**The patient was previously marked to place a total of three trocars: under the axilla (3 mm), at the tip of the scapula for optics (5 mm), and on the anterio-axillary line (3 mm)**.

**Figure 2:**
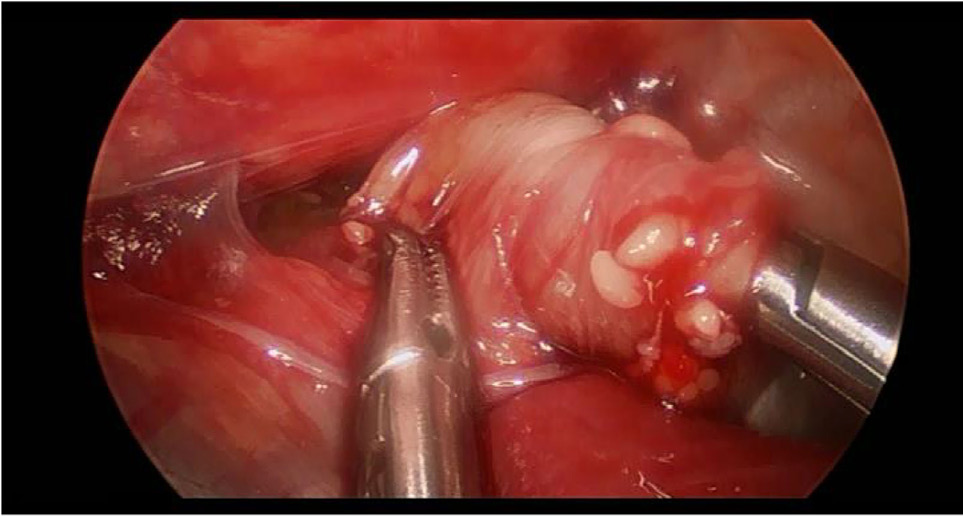
**The figure shows dissection of the upper pouch using “spaghetti maneuver.”** The end of the upper pouch was grasped and, while the operator rolls the grasper, the proximal esophageal pouch was dissected with the other hand.

**Figure 3:**
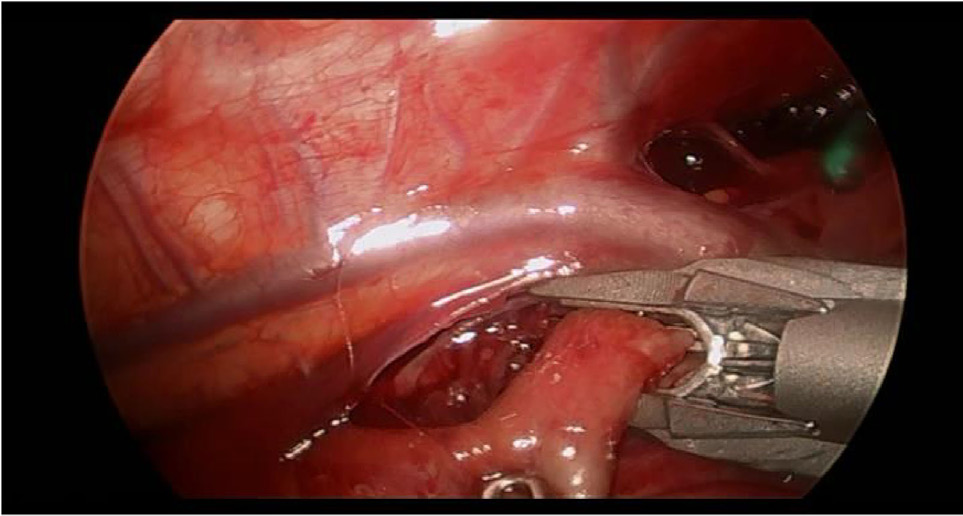
**Tracheoesophageal fistula was approached distally to the arch of the azygos vein**.

**Figure 4:**
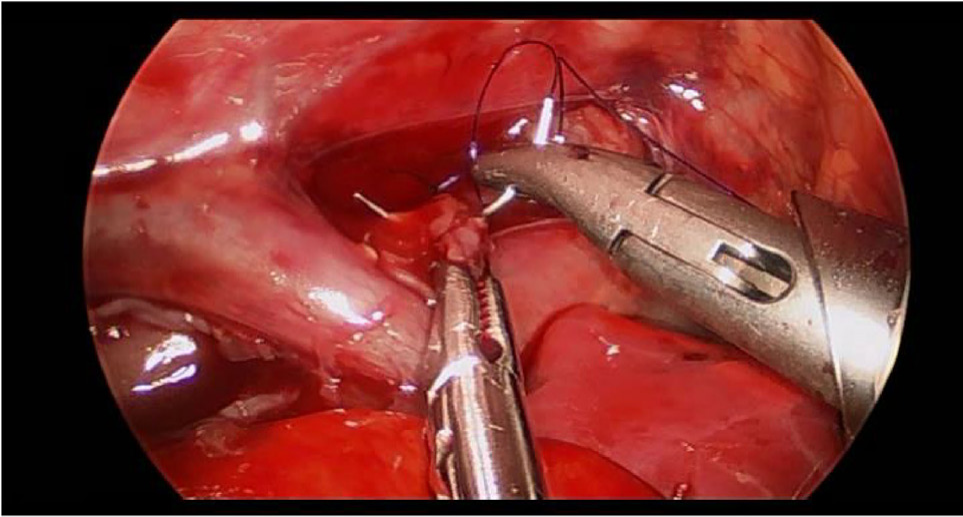
**The first stitch was completed proximal to the arch of the azygos**. All the next procedures were taking place proximally to the azygos vein, leaving the vein untouched.

**Figure 5:**
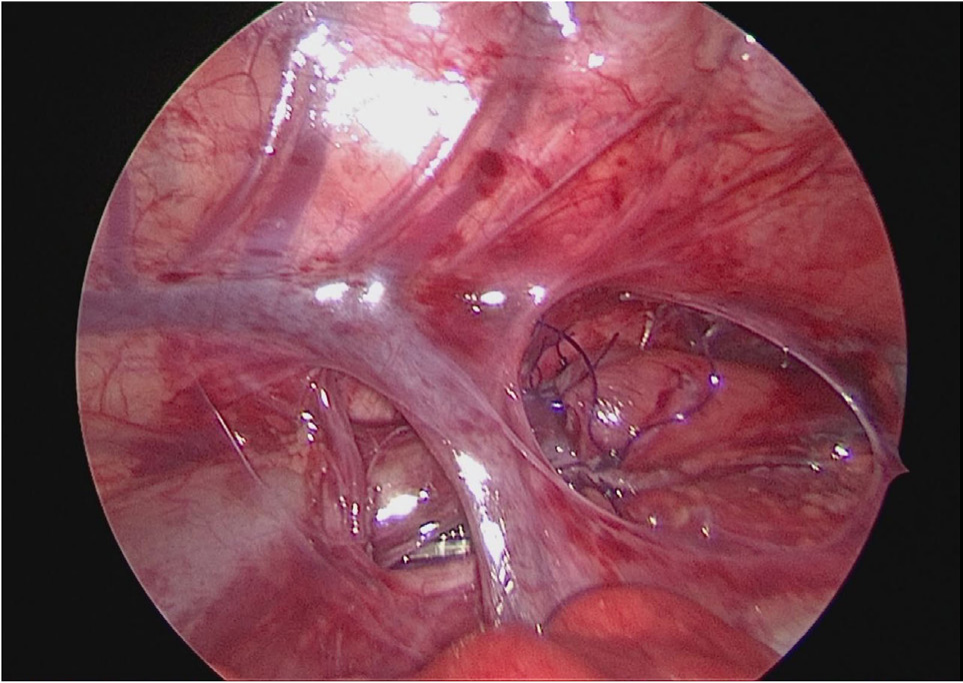
**Final aspect of anatomic thoracoscopic repair of esophageal atresia technique**. Azygos vein was preserved, and a complete esophago-esophageal anastomosis was left in its anatomical place.

Preoperatively, all patients were under endotracheal intubation and general anesthesia. The patients were positioned in Cuschieri position (almost prone position with a slight elevation of the right side of thorax) to access the right hemithorax. Three trocars were placed in triangulation: one under the axilla (3 mm), another at the tip of the scapula for a 30° optics (5 mm), and the other one on the anterio-axillary line (3 mm) (Figure 1). Capnothorax was established with CO_2_ insufflation at 0.5 L/min, up to 5 mmHg of pressure. After identifying the vagus nerve and azygos vein, the TEF was dissected and isolated. The upper pouch was dissected and isolated from the trachea performing the “spaghetti maneuver” (Figure 2). When completely dissected, the upper pouch was widely opened with scissors. A 5-mm clip applier was introduced through the most cranial port, after switching its trocar from 3 to 5 mm. TEF was ligated with two titanium clips and transected distally to the clips (Figure 3). Azygos vein was kept untouched during the entire procedure. The anastomosis was carried out using PDS 5/0. In the particular case of ATREA technique, the first stitch was initiated in the left side of the upper pouch (Figure 4). The lower pouch was positioned medially to the azygos vein in order to achieve an anatomic esophageal position. Two more stitches were performed before introducing the nasogastric tube from the upper to the lower pouch. The following stitches were then easier to carry out, completing a total of around eight stitches. While manipulating the needle, it was essential to be extra careful with the adjacent structures. Finally, the esophago-esophageal anastomosis was inspected to assure the esophageal continuity (Figure 5). A chest tube was left through the most distal port. In Group B, the procedure was similar but anastomosis was performed over (on the right side) of the azygos vein.

Patients were kept ventilated between 2 and 4 days according clinical condition. After that infants were allowed to feed orally.

## Results

Between September 2012 and September 2016, 11 patients with EA and distal TEF were treated by thoracoscopic repair at our center. Seven (4 males) were operated according to ATREA (Group A) and four (1 male) by the classic thoracoscopic repair sparing the azygos vein (Group B).

Data comparing both groups regarding gestational age, gender, karyotype, associated malformations, and birth weight are summarized on Table 1, and there were no statistically significant differences regarding these features.

**Table 1:** **Comparison of patient’s characteristics**.

	Group A (*n* = 7)	Group B (*n* = 4)
**Gender**		
Male	4	1
Female	3	3
**Gestational age (weeks)**		
Median [min–max]	38.7 [37–41]	38.3 [35–39]
**Birth weight (g)**		
Median [min–max]	2,516 [2,250–3,420]	2,623 [2,115–2,960]
**Karyotype changes**	0	0
**Associated anomaly**	4	3

**Figure 6:**
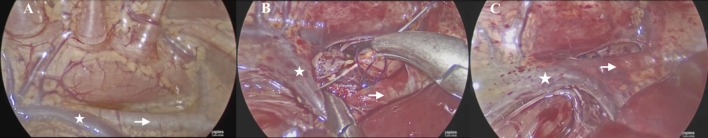
**Anatomic thoracoscopic repair of esophageal atresia technique in a patient with right aortic arch**. **(A)** View before starting dissection; **(B)** anastomosis being done under the azygos vein and aorta; **(C)** final view after anastomosis. *Azigos vein; arrow head indicates aortic vessels.

No karyotype abnormalities identified in both groups. In general, associated anomalies were present in more than 60% of patients (57% of patients from Group A and 75% of patients from Group B). One patient in Group A and two in Group B had VACTERL association. In Group A, there was an infant with a right aortic arc without preoperative diagnosis (Figure 6A), where ATREA was particularly useful as it avoided that the azygos vein and the aortic arch were left compressed in between the esophagus and trachea (Figures 6B,C). All the associated malformations are resumed in Table 2.

**Table 2:** **Associated congenital anomalies in groups A and B**.

	A_1_	A_2_	A_3_	A_4_	B_1_	B_2_	B_3_
**Cardiovascular**			X	X		X	

**Vertebral/skeletal**	X				X	X	

**Genitourinary**		X	X		X		

**Respiratory**							X

**Neurologic**	X					X	X

**Description of associated congenital anomalies**	Scoliosis Block vertebra C5–C6; D10–D11 Tripedicular vertebra L1 Hypoplasia of the corpus callosum	Hypospadias	Right aortic arch Patent ductus arteriosus Persistent left superior vena cava Patent foramen ovale Congenital hypothyroidism Hydronephrosis	Patent foramen ovale	Ureteropelvic junction Obstruction Hydronephrosis Idiopathic localized hydromyelia Scoliosis Supernumerary hemivertebra L4–L5 Tripedicular vertebra L4 Absent intervertebral disc L3–L4	Interventricular communication Interauricular communication Heart failure Thumb hypoplasia (left hand) Hypoplasia of the corpus callosum and septum pellucidum	Ventriculomegaly Agenesis of the septum pellucidum Bronchial malformation

Comparative data regarding surgery and immediate postoperative period and long-term complications are presented in Tables 3 and 4, respectively. The median operative time was 115 [54–135] min in Group A and 151 [123–219] min in Group B with no statistically significant difference.

**Table 3:** **Surgical and immediate postoperative data**.

	Group A (*n* = 7)	Group B (*n* = 4)	Group A vs. Group B
**Age at the time of surgery (days)**
Median [min–max]	3 [3–4]	3.5 [3–5]	*p* = 0.185
**Operative time (min)**
Median [min–max]	115 [54–135]	151 [123–219]	*p* = 0.131
**Surgical complications**	0	0	
**Length of hospitalization (days)**
Median [min–max]	14 [11–28]	56 [15–94]	*p* = 0.088
**Complications during hospitalization**	0	1*-empyema*	*p* = 0.364
**Mechanical ventilation (days)**
Median [min–max]	3 [2–6]	21 [3–52]	*p* = 0.244
**Thoracic drainage (days)**
Median [min–max]	7 [5–9]	9.5 [7–25]	*p* = 0.074
**First feeding (days)**			
Median [min–max]	6 [6–7]	8.5 [6–40]	*p* = 0.072
**Anastomotic leak**	0	1	*p* = 0.364

**Table 4:** **Long-term postoperative data**.

	Group A (*n* = 7)	Group B (*n* = 4)	Group A vs. Group B
**Anastomotic stricture^a^**	3	3	*p* = 0.545
**Number of dilations**	[1–4]	[1–5]	
**Recurrent fistula**	0	0	
**Tracheomalacia**	1	3	*p* = 0.088
**Aortopexy**	0	1	*p* = 0.364
**GERD**	0	2	*p* = 0.109
**Fundoplication**	0	1	*p* = 0.364
**Death**	0	0	

*^a^Requiring at least one dilatation*.

*GERD, gastroesophageal reflux disease*.

In Group A, the median duration of mechanical ventilation was 3 days, the time until first feeds was 6 days, the duration of chest drainage was 7 days, and the median length of hospitalization was 14 days. In Group B, the median duration of mechanical ventilation was 21 days, the time until first feeds was 8.5 days, the duration of chest drainage 9.5 days, and the median length of hospitalization was 56 days, but the median values in Group B are particularly influenced by one patient with severe tracheomalacia that was hospitalized for 94 days.

Group A had three patients with anastomotic stricture requiring at least one dilation and one patient with mild tracheomalacia with no need for aortopexy. No patient had surgical complications, anastomotic leak, recurrent fistula, GERD, need for new surgical correction, or death. Group B had three patients with anastomotic stricture requiring at least one dilation, three with tracheomalacia, one requiring aortopexy and one with GERD demanding fundoplication. There was a case of anastomotic leak, requiring re-intervention. There were no cases of fistula recurrence or death.

## Discussion

Thoracoscopic operations in neonates such as repair of EA and tracheooesophageal fistula can be associated with intraoperative acidosis and hypercapnia in the absence of hypoxia. These derangements in intraoperative gas exchanges seem to be related to the insufflation and absorption of medical CO_2_ with unknown effects on the developing brain (9). While this is not completely clarified, thoracoscopic technique has been adopted worldwide, by several centers, as the first line approach to EA correction.

Thoracoscopic technique was based on the thoracotomy procedure, as it follows its main steps, including azygos vein ligation and division to optimize the approach to the esophageal pouches.

The excellent visualization of the surgical field provided by thoracoscopy turned the division of the azygos vein practically unnecessary and even questionable. As already reported by others, azygos vein is a major vessel of the human body which means that it should be preserved whenever it is possible (10). Previous studies reported the potential benefits of saving azygos vein in the thoracotomy repair. Evans et al. reported a lifesaving case by preserving the azygos vein in a newborn with EA and an interrupted inferior vena cava (IVC) (13). Although this is a rare combination, we know that newborns with EA have increased risk of cardiovascular malformations. In the particular case of an interrupted IVC, dividing the azygos vein would be catastrophic (13). A prospective study of newborns with EA and TEF proposed that preservation of azygos vein offers an additional protection to prevent anastomotic leaks (10). The preservation of the azygos vein avoids early postoperative local edema, offering a protective factor against anastomotic leaks. Another study showed a decreased rate of pneumonitis in the postoperative period when preserving the azygos vein (11). Patkowski et al. reported a series of 23 patients with EA and TEF corrected by thoracoscopy with preservation of azygos vein (12). Differently to ATREA, they separated the anastomosis from the fistula ligation interposing azygos vein between them. With this technique, the authors argued that azygos may prevent recurrent fistula formation (12). This might be true; anyway, the entrapment of the azygos vein between the trachea and esophagus and the friction resulting from the esophagus movements could lead to a variable degree of collapse of the azygos vein, probably making it useless or at least with a major impact on its hemodynamics.

It is our belief that the best approach would be to preserve the normal anatomophysiology of the human body, by reconstructing the esophagus on its anatomic position, medial to the azygos vein. This possibility became feasible in a newborn in whom the TEF was located in a high position of the trachea; therefore, it was almost intuitive to perform the correction by ATREA technique. In this case, we were able to safely finish the anastomosis with no peroperative or postoperative complications. Subsequently, we have attempted to apply the ATREA to every newborn with the diagnosis of EA with distal fistula, and we have succeeded in a total of six patients. The exception was a case in whom the anastomosis was under a great tension and we decided to use the classical thoracoscopic technique for safety reasons. With experience, we could verify that thoracic vessels (azygos vein and aortic branches) are sufficiently elastic to allow dissection around and/or under them. By doing this, we predict we can perform ATREA in the great majority of cases. Actually, our short series could document that ATREA has shown no difference in terms of operative details, hospitalization, and follow-up as compared with the classic thoracoscopic repair sparing azygos vein. Therefore, we can conclude that ATREA is feasible and likely a safe technique.

Additionally, the principles used in ATREA can also be applied in cases of venous return malformations and even in cases of arterial malformations such as aortic arch malformations. In fact, we had a patient in Group A, where we unexpectedly discovered a right aortic arch. In this case, we did not need to do an immediate left thoracoscopy neither to postpone the repair as suggested by a recent systematic review (14). Actually, we could perform dissection and anastomosis in anatomic position. The experience with this patient provides additional evidence that surgeons might be prepared to try anatomic repair in all patients as this experience could be useful when unexpected major aortic malformations appears, including those patients that are still operated by open/thoracotomy access.

The authors recognize the limitations of this study, namely a small sample size and the fact that the all patients were operated on by the same surgeon. It may be arguable that an extensive surgical expertize may influence the results positively; nevertheless, a single surgeon series also eliminates any inter-surgeons variability. So, larger studies or randomized clinical trials are necessary to understand all the benefits or potentially disadvantages, of such a modification. Evaluation of the patency and hemodynamics of the azygos vein after both classic and ATREA repair could also clarify the impact of the surgical approach on such vessel.

## Ethics Statement

This study was submitted and approved by the following ethics committees: “Comissão de Ética para o Hospital de Braga (ref. CESHB 087/2016)” and “Subcomissão de Ética para as Ciências da Vida e da Saúde (ref. SECVS 015/2016).”

## Author Contributions

Substantial contributions to the conception or design of the work or the acquisition, analysis, or interpretation of data for the work; drafting the work or revising it critically for important intellectual content; final approval of the version to be published; and agreement to be accountable for all aspects of the work in ensuring that questions related to the accuracy or integrity of any part of the work are appropriately investigated and resolved: JF, CB, RL-P, AS, and JC-P.

## Conflict of Interest Statement

The authors declare that the research was conducted in the absence of any commercial or financial relationships that could be construed as a potential conflict of interest.
